# Case report: Lingual dystonia symptoms treated with botulinum toxin in patients with THAP1 mutation

**DOI:** 10.3389/dyst.2023.11361

**Published:** 2024-01-04

**Authors:** Aparna Wagle Shukla, Nicole Herndon, Irene Malaty

**Affiliations:** 1 Department of Neurology, Fixel Institute for Neurological Diseases, University of Florida, Gainesville, FL, United States; 2 UF Health Rehab Center, Fixel Institute for Neurological Diseases, University of Florida, Gainesville, FL, United States; 3 Department of Speech, Language, and Hearing Sciences, University of Florida, Gainesville, FL, United States

**Keywords:** lingual dystonia, oromandibular dystonia, botulinum toxin, THAP1, DYT-THAP1

## Abstract

**Background:**

THAP1 mutation dystonia is a known genetic cause of generalized dystonia. THAP1 mutation frequently presents with clinical features of bulbar dysfunction, including oromandibular and lingual dystonia. Patients complain of significant speech, chewing, and swallowing difficulties leading to major occupational and social disabilities. While bilateral globus pallidus internus deep brain stimulation (DBS) is powerful therapy for generalized dystonia and improves dystonia symptoms in the cervical and limb region, it may not improve speech despite multiple adjustments to the stimulation parameters. Treating lingual dystonia symptoms with oral medications is commonly unsatisfactory. Botulinum toxin injection, a potent therapy for focal forms of dystonia is currently underutilized in clinical practice for treating lingual dystonia.

**Cases:**

We present two patients with THAP1 mutation reporting lingual dystonia symptoms. The first patient did not meet the eligibility criteria for DBS therapy due to significant psychiatric symptoms. The second patient received DBS with improvements in cervical, limb, and trunk symptoms but complained of severe speech difficulties that did not improve despite multiple programming sessions. These patients were treated with botulinum toxin injections every 12 weeks for more than 3 years, with speech improvements lasting most of the cycle. For the most part they tolerated botulinum toxin without bothersome side effects. Along with the clinical histories, we present objective perceptual analysis of speech samples collected before and after botulinum toxin injections in one of the treatment cycles.

**Conclusion:**

Botulinum toxin injections that are clinically beneficial for mitigating lingual dystonia symptoms should be utilized to address symptoms of THAP1 mutation dystonia that may not be amenable to other therapies, such as the DBS.

## Introduction

DYT-*THAP1* is a frequent familial cause of dystonia due to mutations in the THAP1 gene (THAP domain-containing, apoptosis-associated protein 1). DYT-*THAP1* is an autosomal dominant disorder with generalized or segmental dystonia symptoms [1]. The clinical symptoms can appear from childhood through adulthood, with the average age of onset being 18 years [2]. The phenotype is highly variable even within a single family ranging from unaffected carriers to generalized dystonia. The clinical symptoms are often prominent in the cervical, bulbar and oromandibular regions (including the tongue) [3, 4]. Patients with lingual dystonia complain of significant speech, chewing, and swallowing difficulties which leads to occupational and social disability [5].

Deep brain stimulation (DBS) targeted to the globus pallidus internus (GPi) is considered for treating generalized distribution symptoms of DYT-*THAP1* dystonia that are frequently refractory to oral medications [6]. However, the DBS outcomes are frequently suboptimal and unsatisfactory [7, 8] as the speech and swallowing difficulties related to lingual dystonia may not improve [7, 9, 10]. Sometimes, DBS can lead to an undesirable worsening of speech due to the spread of stimulation to unintended brain regions or fibre tracts, for example, stimulation of the corticospinal fibres coursing medially and posteriorly to posteroventral GPi. Thus, improving all aspects of DYT-*THAP1* dystonia can be challenging for physicians and frustrating for patients.

We present two cases of DYT-THAP1 that manifested with symptoms of lingual dystonia, leading to profound speech difficulties interfering with daily communications. The first patient was deemed ineligible for the DBS surgery as he had significant psychiatric symptoms. The second patient continued to have significant lingual dystonia despite multiple trials of stimulation settings in varying combinations. However, upon receiving botulinum toxin injections targeted to the lingual muscles, these patients began to endorse improvements in speech. We present the clinical data, including the findings of a perceptual analysis performed on their speech samples before and after botulinum therapy.

### Patient 1

A 49 years-old left-handed white male with a genetically confirmed diagnosis of DYT-*THAP1* dystonia with mutation identified as c.197_196delAG (p.E66VfsX19), presented to our center with the following history. His birth and developmental history were unremarkable. When he was 7 years old, he began to have motor difficulties and abnormal movements. His left hand began to rotate involuntarily and assume an abnormal posture when writing, eating, or playing with toys. At age 18, his head began to involuntarily turn to the left, especially when driving or watching television. He observed that the neck pulling was accompanied by mild to moderate shaking. He experienced some relief when he held his chin in a specific position. At age 39, he began to notice involuntary tongue and jaw closure movements triggered mainly by speaking and chewing. His tongue involuntarily protruded and rolled in his mouth when he had conversations with his friends and family. The tongue movements also interfered with chewing and swallowing. He began to experience copious drooling and repeated episodes of tongue-biting. His chewing and swallowing difficulties became so pronounced that he began to take small portions of a puree diet. He felt his tongue was growing in size due to repetitive movements, which further aggravated his condition. He sometimes had to manually push his tongue back in the mouth when it protruded, place a towel to bite down or insert a straw in the mouth to obtain relief. He found communicating with friends and family nearly impossible, especially in the afternoon and evening.

**FIGURE 1:**
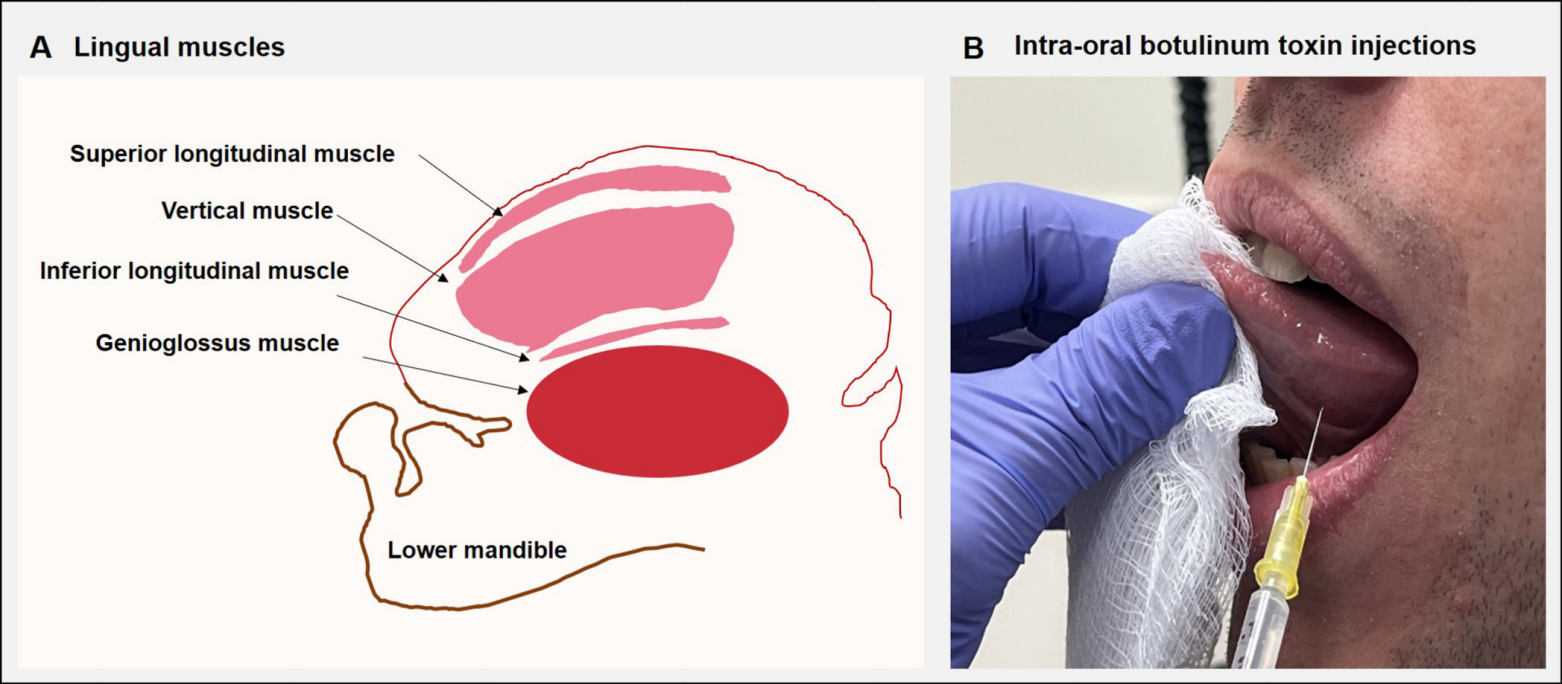
**(A)** The figure illustrates the sagittal section of the tongue, the muscles of the tongue, and the lower jaw. The superior longitudinal muscle runs beneath the mucosa of the dorsum of the tongue, shortens the tongue, and turns the apex and sides upwards. The inferior longitudinal muscle is close to the inferior lingual surface and draws the apex downwards to make the dorsum convex. The vertical muscle passes from the dorsal to the ventral aspects of the tongue in the anterior borders; the muscle flattens and widens the tongue. The genioglossus provides traction to move the tongue forwards to protrude its apex from the mouth. **(B)** The patient was asked to keep the mouth open as wide as possible and attempt to voluntarily protrude the tongue slightly, pointing upwards towards the upper lip. A disposable hypodermic 1.5-inch 30G needle was inserted intraorally into the targeted muscles (mainly vertical, inferior longitudinal, and genioglossus). The figure shows targeting of vertical muscle. No local anesthesia was needed as the patients tolerated the procedure. Once the hypodermic needle was inserted, aspiration was done to ensure there was no blood; subsequently, planned doses of the botulinum toxin were injected into the target muscles. EMG guidance was not used for the injection procedure.

As a consequence, he felt recluse, withdrawn, lonely and depressed. Physical examination revealed severe tongue curling and protrusion. He underwent treatment trials with levodopa and anticholinergic drugs with no clinical improvements. During the multidisciplinary evaluation, he was deemed ineligible for DBS surgery due to significant anxiety and depression. He began to receive intraoral onabotulinum toxin injections into the genioglossus, inferior longitudinal, and vertical muscles, which led to noticeable speech improvements. In the beginning, low doses of 5 units were used to minimize the risk of side effects. Within two cycles, doses were titrated up to 15 units on each side and injected into the genioglossus (10 units), inferior longitudinal muscle (2.5 units), and vertical muscles (7.5 units) (Figure 1). He began to report improved communication abilities with titration of doses to 40 units/cycle. The benefits were noted about 2 weeks after injections and lasted around 6 weeks. He has received onabotulinum toxin injections every 12 weeks for the last 4 years, with persistent speech improvements. Only in two of the injection cycles he reported transient worsening of swallowing that lasted for 3 weeks. In one of the recent injection cycles, speech samples were recorded before and 4 weeks after injections (Supplementary Audio S1). The patient was instructed to recite the standardized “rainbow passage” in a quiet room with the recorder held 10 cm from the patient’s mouth. The “rainbow passage,” considered phonetically balanced for the English language, helps assess speech. He was also instructed to recite the /p/, /t/, and /k/ syllables during the diadochokinesis (DDK) task. The following speech parameters, including intelligibility, naturalness (intonation, intensity, rhythm), rate, volume, prosody, articulatory precision, vocal quality, resonance, and presence of tremor, were perceptually analyzed by a speech pathologist blinded to the time of data collection. Improvements were perceived in speech intelligibility, articulatory precision, and vocal quality (marked by asterisks). Table 1 illustrates the findings.

### Patient 2

A 61 years-old right-handed white male presented with a diagnosis of genetically confirmed DYT-THAP1 dystonia with mutation identified as c.331C>T (p.Gln111*) and a history of bilateral GPI DBS surgery. He first developed symptoms at the age of 16 years. His left arm began to involuntarily twist and turn, which soon spread to the right arm, impacting his day-to-day functioning. Within 2 years, his head began to turn to the right, and his trunk arched backward. His gait became slowly awkward as the legs began to turn in involuntarily. At age 25, he began to have speech difficulties. His tongue was observed to protrude and twist involuntarily, resulting in poorly intelligible speech. He complained of mild tightening of the jaw muscles, difficulty chewing, and copious drooling of saliva. He found little improvements with clonazepam, cyclobenzaprine, and anticholinergic drug therapy. At age 40, he underwent bilateral GPi DBS surgery, and within 8 months, he began to endorse improvements in his arm symptoms. While the neck, trunk, and gait symptoms needed an additional 5 months of DBS programming, the speech and swallowing remained affected, with substantial communication difficulties. He found his tongue involuntarily rolling, curling, and protruding whether at rest or during speaking. He frequently had spillage through the lips when eating food and drinking liquids. He complained that his tongue felt like a large object in his mouth. Physical examination confirmed tongue protrusion and twisting interfering significantly with speech. He began to receive onabotulinum toxin injections administered intraorally to the lingual muscles. The dose initiated at 10 units was titrated up to 40 units per session with genioglossus (10 units), vertical muscle (5 units), and inferior longitudinal muscle (5 units). With these doses, he began to find communications relatively more effortless than before. His speech was noted to be comprehensible when communicating with single words and short phrases. He complained of worsening swallowing when the dose was further escalated to 60 units. He received botulinum toxin for the subsequent 3 years, with benefits persisting for 8 weeks out of 12 weeks. A perceptual analysis found that intelligibility, naturalness, articulatory precision, and vocal quality improved at the peak dose effects (around 4 weeks after injections). Table 1 illustrates improvements marked by asterisks.

## Discussion

We present speech outcomes of DYT-*THAP1* mutation patients presenting with pronounced lingual dystonia symptoms that responded to botulinum toxin injections. While the first patient was deemed ineligible for DBS surgery as he had significant anxiety and depression, the second patient, despite multiple programming sessions, did not perceive improvements in lingual dystonia. Botulinum toxin injections to the tongue muscles relieved symptoms that positively impacted speech and communication.

Lingual dystonia, a form of oromandibular dystonia, is relatively rare, representing 3%–5% of all dystonia. Lingual dystonia patients experience difficulties with chewing, swallowing, speaking. The patients complain of social embarrassment, depression, weight loss, and reduced quality of life [5, 11]. In severe cases, lingual dystonia is associated with self-mutilating life-threatening behavior, including tongue biting and lip biting [12]. The lingual movements are episodic and repetitive or constant and sustained. Four subtypes of lingual dystonia are recognized including protrusion, retraction, curling, and laterotrusion [13]. DYT-*THAP1* often presents with lingual dystonia. Chewing gum, switching the type of food, touching jaws with a finger or hand, or using a mask are sensory tricks that might temporarily alleviate symptoms [14]. Other sensory tricks include using a pipe, wood, straw, toothpick, and cotton. Some studies have found partial improvements using removable splints such as occlusal splints and dental devices [15].

DYT-*THAP1* patients often require surgical interventions with DBS as the anticholinergic drugs, muscle relaxants, and muscle afferent blocks have limited success. Although many studies have found bilateral GPi DBS to improve limb and cervical dystonia, most series report limited or no improvement in speech or bulbar function [8]. In one of the largest series published thus far, 14 patients with lingual dystonia treated with bilateral GPi DBS had around 50% improvement at a median follow-up of 4 years. However, the effect was remarkably greater for dystonia affecting the trunk and limbs than the neck, and orolaryngeal regions [10].

There is limited data on the clinical outcomes of botulinum toxin injection therapy in lingual dystonia [16]. The earliest case series was published over three decades ago. Although botulinum toxin led to nearly 50% improvements, many patients developed dysphagia leading to secondary aspiration pneumonia and breathing difficulties [17]. The tongue is relatively difficult to target with botulinum toxin injection. Some potential consequences are dysphasia, dysarthria, masticatory disturbance, and breathing difficulties [13]. Some injectors employ submandibular [18] route and some apply the intraoral route for injections [16, 19]. In one of the largest cohorts that comprised of protrusion (68.6%), retraction (16.9%), curling (7.6%), and laterotrusion (7.0%) subtypes, botulinum toxin injections were administered into the genioglossus and/or intrinsic muscles via individualized submandibular and/or intraoral routes. Overall there was a 70% improvement in mastication, speech, pain, and discomfort [13]. The curling type showed the greatest improvement (81.9%), while the retraction type showed the least improvement (67.9%). Mild and transient dysphasia occurred in 12.5% of patients but disappeared spontaneously within several days to 2 weeks. No serious side effects were observed. Our cases were different from these case series in various ways. Most patients in the case series had tardive task-specific symptoms, and none reported a genetic etiology. Our patients had symptoms regardless of whether the tongue was at rest or in action. Our cases provide long-term follow-up data on speech outcomes that were not available in the literature.

In summary, our cases illustrate that botulinum toxin could improve speech in patients with DYT-THAP1 mutation, and the benefits could be sustained for a long time. Our patients endorsed speech improvements for more than 12 consecutive injection cycles with no major side effects. Although more data with long-term blinded follow-up will be needed, our study findings imply that botulinum toxin should be utilized to address some aspects of DYT-THAP1 generalized dystonia that may not be amenable to other therapies such as the DBS.

## Supplementary Material

Patient 1 Speech SUPPLEMENTARY AUDIO S1
A speech pathologist blinded to the time of assessment analyzed the speech recordings. The recording includes a speech sample for patient 1. The first 55 s includes the “rainbow passage” recitation and diadochokinesis (DDK) task for /p/, /t/, /k/ syllables before the patient received botulinum toxin injections. At the recording time, at least 12 weeks had elapsed after the last round of botulinum injection, and the patient reported that the clinical benefits had completely worn off. The second part of the recording that can be heard after a pause of 5 s represents the repeat recitation of the “rainbow passage“ and /p/, /t/, /k/ syllables performed 4 weeks after receiving the botulinum toxin injections.

Patient 2 speech SUPPLEMENTARY AUDIO S2
A speech pathologist blinded to the time of assessment analyzed the speech recordings. The recording includes a speech sample for patient 2. The first 98 s of the speech sample represents “rainbow passage” and /p/, /t/, /k/ recited before receiving botulinum toxin injections. The recording was performed 12 weeks after the last round of botulinum injection. The patient’s DBS was kept on at the time of recording. The second part of the recording that can be heard after a pause of 10 s represents the same “rainbow passage” and /p/, /t/, /k/ recited 4 weeks after receiving botulinum toxin injections.

## Figures and Tables

**TABLE 1:** Perceptual speech assessment of patients before and after botulinum toxin injections.

	Pre botulinum	Post botulinum

Patient 1		

Intelligibility	Severe	Moderate*
Naturalness	Moderate to severe	Mild to moderate*
Rate of speech	Very mildly slow	Mildly slow
Volume	Normal	Normal
Prosody	Variable	Variable
Articulatory precision during passage reading and diadochokinesis (DDK)	Passage reading severely imprecise; DDK moderately imprecise	Passage reading moderately imprecise; DDK mild improvement in /p/*
Vocal quality	Mild-to-moderately harsh	Minimally harsh*
Resonance	Hypernasality	Hypernasality
Tremor	Absent	Absent

Patient 2		

Intelligibility	Severe	Moderate-to-severe
Naturalness	Severe	Moderate-to-severe
Rate of speech	Moderately slow	Moderately slow
Volume	Normal	Normal
Prosody	Variable; monotone	Variable; monotone
Articulatory precision during passage reading and DDK	Passage reading profoundly imprecise DDK profoundly imprecise	Passage reading slightly improved; DDK for /p/ and /t/ significantly improved*
Vocal quality	Severely strained and harsh	Moderately harsh and wet*
Resonance	Hypernasality	Hypernasality
Tremor	Absent	Absent

* denotes a significant improvement..

## Data Availability

The original contributions presented in the study are included in the article/Supplementary Material, further inquiries can be directed to the corresponding author.
